# *SMOC2* gene interacts with *APOL1* in the development of end-stage kidney disease: A genome-wide association study

**DOI:** 10.3389/fmed.2022.971297

**Published:** 2022-09-28

**Authors:** Ninad S. Chaudhary, Nicole D. Armstrong, Bertha A. Hidalgo, Orlando M. Gutiérrez, Jacklyn N. Hellwege, Nita A. Limdi, Richard J. Reynolds, Suzanne E. Judd, Girish N. Nadkarni, Leslie Lange, Cheryl A. Winkler, Jeffrey B. Kopp, Donna K. Arnett, Hemant K. Tiwari, Marguerite R. Irvin

**Affiliations:** ^1^Department of Epidemiology, University of Alabama at Birmingham, Birmingham, AL, United States; ^2^Department of Epidemiology, Human Genetics, and Environmental Sciences, School of Public Health, Human Genetics Center, University of Texas Health Science Center at Houston, Houston, TX, United States; ^3^Department of Medicine, University of Alabama at Birmingham, Birmingham, AL, United States; ^4^Division of Genetic Medicine, Department of Medicine, Vanderbilt Genetics Institute, Vanderbilt Epidemiology Center, Vanderbilt University Medical Center, Nashville, TN, United States; ^5^Department of Neurology, University of Alabama at Birmingham, Birmingham, AL, United States; ^6^Division of Clinical Immunology and Rheumatology, Department of Medicine, University of Alabama at Birmingham, Birmingham, AL, United States; ^7^Department of Biostatistics, University of Alabama at Birmingham, Birmingham, AL, United States; ^8^Division of Data-Driven and Digital Medicine (D3M), Icahn School of Medicine at Mount Sinai, New York, NY, United States; ^9^Department of Medicine, University of Colorado Denver - Anschutz Medical Campus, Denver, CO, United States; ^10^Basic Research Program, National Cancer Institute, National Institutes of Health, Frederick National Laboratory for Cancer Research, Frederick, MD, United States; ^11^National Institute of Diabetes and Digestive and Kidney Diseases, National Institutes of Health, Bethesda, MD, United States; ^12^Deans Office, College of Public Health, University of Kentucky, Lexington, KY, United States

**Keywords:** *APOL1*, gene–gene interaction, SMOC2, kidney disease, end-stage kidney disease, genome-wide analysis, African-Americans

## Abstract

**Background:**

Some but not all African-Americans (AA) who carry *APOL1* nephropathy risk variants (*APOL1*) develop kidney failure (end-stage kidney disease, ESKD). To identify genetic modifiers, we assessed gene–gene interactions in a large prospective cohort of the REasons for Geographic and Racial Differences in Stroke (REGARDS) study.

**Methods:**

Genotypes from 8,074 AA participants were obtained from Illumina Infinium Multi-Ethnic AMR/AFR Extended BeadChip. We compared 388 incident ESKD cases with 7,686 non-ESKD controls, using a two-locus interaction approach. Logistic regression was used to examine the effect of *APOL1* risk status (using recessive and additive models), single nucleotide polymorphism (SNP), and *APOL*1^*^SNP interaction on incident ESKD, adjusting for age, sex, and ancestry. APOL1^*^SNP interactions that met the threshold of 1.0 × 10^−5^ were replicated in the Genetics of Hypertension Associated Treatment (GenHAT) study (626 ESKD cases and 6,165 controls). In a sensitivity analysis, models were additionally adjusted for diabetes status. We conducted additional replication in the BioVU study.

**Results:**

Two *APOL1* risk alleles prevalence (recessive model) was similar in the REGARDS and GenHAT studies. Only one *APOL1*–SNP interaction, for **rs7067944** on chromosome 10, ~10 KB from the *PCAT5* gene met the genome-wide statistical threshold (*P*_interaction_ = 3.4 × 10^−8^), but this interaction was not replicated in the GenHAT study. Among other relevant top findings (with *P*_interaction_ < 1.0 × 10^−5^), a variant (**rs2181251**) near *SMOC2* on chromosome six interacted with *APOL1* risk status (additive) on ESKD outcomes (REGARDS study, *P*_interaction_ =5.3 × 10^−6^) but the association was not replicated (GenHAT study, *P*_interaction_ = 0.07, BioVU study, *P*_interaction_ = 0.53). The association with the locus near *SMOC2* persisted further in stratified analyses. Among those who inherited ≥1 alternate allele of rs2181251, *APOL1* was associated with an increased risk of incident ESKD (OR [95%CI] = 2.27[1.53, 3.37]) but *APOL1* was not associated with ESKD in the absence of the alternate allele (OR [95%CI] = 1.34[0.96, 1.85]) in the REGARDS study. The associations were consistent after adjusting for diabetes.

**Conclusion:**

In a large genome-wide association study of AAs, a locus *SMOC2* exhibited a significant interaction with the *APOL1* locus. *SMOC2* contributes to the progression of fibrosis after kidney injury and the interaction with *APOL1* variants may contribute to an explanation for why only some *APOLI* high-risk individuals develop ESKD.

## Introduction

Two risk variants in the *APOL1* gene on chromosome 22, collectively referred to as *APOL1* nephropathy risk alleles are associated with an increased risk of chronic kidney disease, and kidney failure (end-stage kidney disease, ESKD) among self-reported African-American (AA) individuals (1, 2). However, these variants are not completely penetrant for kidney disease incidence or progression, and a better understanding of the role of modifying environmental and/or genetic factors are needed (3, 4). The extent of molecular interactions in gene regulation and metabolic systems suggests that the interactive relationship between DNA variants can better explain the biological underpinnings of clinical endpoints than analysis based only on the variants.

Previous studies have investigated the role of various single nucleotide polymorphisms (SNPs) and the *APOL1* risk variants in kidney diseases (5–7). Bostrom et al. (5) performed a case-control association study of 1,420 SNPs in 962 AA non-diabetic nephropathy cases and 932 AA non-nephropathy controls. They found six SNPs that met an interactive *p*-value threshold of 0.001 with *APOL1* variants, under recessive, additive, or dominant models (5). Divers et al. (6) expanded these findings in a larger sample size of 1,367 AA non-diabetic ESKD patients and 1,504 related controls using a similar pooled set of SNPs specific to kidney diseases. The study examined interactions among the top 42 genes identified in the main effects association analysis, and found a variant, rs16854341, in the podocin gene to be of particular importance (6). Although the variant did not meet the genome-wide significant threshold, the presence of this variant reduced the odds of developing ESKD due to *APOL1* risk variants. These studies focused on populations without diabetes and identified only genes relevant to non-diabetic nephropathy.

In the present study, using genotype data from 8,074 AA participants from a community sample of the REasons for Geographic and Racial Differences in Stroke study (REGARDS), we tested whether SNPs from GWAS modify the association of *APOL1* risk variants with ESKD in a different AA cohort. Genotypic data was obtained from a contemporary 1.4 million SNPs multi-ethnic genotype array, which was further enriched for extensive new African variant coverage. The findings were replicated in 6,791 AA from the Genetics of Hypertension Associated Treatment (GenHAT) study using the same array.

## Methods

### Discovery population

The REGARDS study is one of the largest ongoing prospective population-based studies in the U.S. and was designed to measure stroke incidence and associated risk factors in AA and European-Americans adults ≥45 years of age (8). From January 2003 to October 2007, 30,239 participants (42% AA, 55% women) were recruited from the 48 contiguous U.S. states and the District of Columbia. At baseline, demographics, medical history, and clinical data were obtained via telephone and an in-home visit. Blood and urine samples were also obtained at the baseline in-home visit. Participants were subsequently contacted every 6 months by telephone to assess data on new-onset stroke, coronary heart disease, and death which was further adjudicated by retrieving the pertinent medical records. Additionally, a linkage with United States Renal Dialysis System (USRDS) was established to identify participants who may have developed ESKD during the follow-up (9). After limiting the participants to those with known ESKD status and *APOL1* status as well as meeting the quality control standards of genotyping (see below), the data on 8,074 AA participants were available for analysis.

### Replication populations

Genetics of Hypertension Associated Treatment is a pharmaco-genetic ancillary study to the Anti-hypertensive and Lipid-Lowering Treatment to Prevent Heart Attack Trial (ALLHAT), designed to identify the genes associated with anti-hypertensive treatment response that can potentially modify the risk of cardiovascular outcomes (10). Anti-hypertensive and Lipid-Lowering Treatment to Prevent Heart Attack Trial was the largest randomized, double-blind multi-center anti-hypertensive clinical trial, and included persons ≥55 years with hypertension and at least one cardiovascular risk factor (11). Participants were evaluated at 3, 6, 9, and 12 months during the first year, and every 4 months thereafter to monitor adherence to the treatment plan and to collect clinical data and blood and urine samples. Data on estimated glomerular filtration rate and ESKD were obtained as secondary outcomes. The present analytical sample consisted of 6,791 AA participants who met the inclusion criteria described above for the REGARDS study. Additional replication at one locus (*SMOC2*) was conducted in the Vanderbilt Biobank of DNA (BioVU DNA) repository. The BioVU DNA Repository is a deidentified database of electronic health records (EHR) that are linked to patient DNA samples at Vanderbilt University Medical Center. A detailed description of the database and how it is maintained has been published elsewhere (12).

### Genotyping

Genome-wide genotyping was performed within each study independently using Illumina Infinium Multi-Ethnic AMR/AFR BeadChip Arrays (MEGA chip; Illumina, San Diego, CA). Similar imputation and quality control procedures were implemented for REGARDS and GenHAT study groups. Briefly, data were imputed using the NHLBI TOPMed release 2 reference panel (Freeze 8) using the TOPMED Imputation server developed at the University of Michigan (13, 14). Around one million variants in the REGARDS study and 970k variants in the GenHAT study were imputed. Samples with call rates <95%, internal duplicates, or sex mismatches were removed. Ancestry information was obtained using principal component analysis in the EIGENSTRAT program (15, 16). Individuals who were outliers for ancestry (more than six standard deviations) were removed. Approximately 21 million variants were available for association analysis with imputation quality scores (MACH *r*^2^) ≥0.3 and minor allele count ≥20 in both the REGARDS and GenHAT populations. Quality control for the BioVU cohort included excluding samples or variants with missingness rates above 2%. Samples were also excluded if consent had been revoked, a sample was duplicated, or failed sex concordance checks. The data for BioVU was also imputed using the NHLBI TOPMed release 2 reference panel (Freeze 8).

### *APOL1* genotyping

*APOL1* risk variants consist of two missense mutations (rs73885319 and rs60910145) (together labeled as G1), and one 6-bp deletion (rs71785313; labeled as G2). The G1 risk alleles are 128 bp apart and are in perfect or almost perfect disequilibrium representing the G1 haplotype (1, 17, 18). These variants were directly identified in the REGARDS study using TaqMan SNP Genotyping Assays in prior projects (19, 20). The number of *APOL1* risk alleles was recorded as two copies if participants had either G1/G1, G1/G2, or G2/G2 and one copy if participants had G1/G0 or G2/G0. The state of G0/G0 indicates absence of both G1 and G2 variants. The primary genetic inheritance model was additive, such that each risk allele conferred additional risk while the secondary genetic inheritance model was recessive so that those with zero or one copy were compared to those with two copies. Data for *APOL1* variants in the GenHAT study were obtained using the genotypic array data. The genotyped data on the rs143830837 variant (hg38 base pair, 36265995) was available in GenHAT instead of rs71785313 (hg38 base pair 36265996). Single nucleotide polymorphism (SNP) rs14383087 is merged with rs71785313 in subsequent assembly as reported in the NCBI dbSNP database (https://www.ncbi.nlm.nih.gov/snp/?term=rs143830837) and are the same variants. BioVU *APOL1* was assessed using variants directly genotyped on the MEGA array (only G2 and one of the G1 SNPs). The imputation of both G1 SNPs was evaluated and had 99.9% concordance with the directly genotyped G1 variant.

### Outcomes

The main study outcome was incident ESKD, identified using an existing linkage of the REGARDS study with USRDS data accessed through December 2019 (9). The USRDS is a comprehensive national registry that collects, analyzes, and distributes information on the ESKD population in the U.S., including treatment failure and mortality (21). For the REGARDS study with available *APOL1* data, there were 388 incident ESKD events retrieved via the USRDS link. In the GenHAT study, incident ESKD (*n* = 128) was recorded if the participant started on dialysis or had a kidney transplant. In the BioVU study, ESKD was identified using diagnostic (ICD 9 and 10) codes for dialysis (2 or more instances of codes V45.11 or Z99.2), kidney transplant (2 or more instances of codes V42.0 or Z94.0), and ESKD (5 or more instances of codes 585.6 or N18.6) across the entire available deidentified electronic medical record.

### Covariates

Information on age, sex, and diabetes status was obtained from baseline visits in both studies. Diabetes mellitus in the REGARDS study was defined as fasting serum glucose ≥126 mg/dl, non-fasting serum glucose ≥200 mg/dl, or use of glucose-lowering medication. Diabetes mellitus in the GenHAT study was defined as fasting serum glucose >140 mg/dl, or non-fasting serum glucose >200 mg/dl in the past 2 years, and/or use of injected or oral insulin or oral hypoglycemic agents. In BioVU EHR data, age was assigned as the earliest outcome code age for cases, and the age at the end of their medical record for controls (i.e., the latest age at which they were determined to be ESKD-free). Diabetes status for the BioVU study was assigned based on ICD codes, and hypertension status was defined as taking anti-hypertension medications, having two or more ICD codes for hypertension, or having two or more outpatient blood pressures >140/90.

### Statistical analysis

Baseline characteristics of both the REGARDS and GenHAT population were tabulated. PLINK2 software was used to perform logistic regression for incident ESKD outcomes, including a term for two-locus interaction (22). For this approach, *APOL1* risk status was analyzed under the additive model and the recessive genetic model (binary variable). Each GWAS SNP was analyzed under an additive genetic model such that each analytical model consisted of *APOL1* risk status, SNP, *APOL*1^*^SNP, age, sex, and principal components of ancestry. The genome-wide threshold for significant interaction was *P*_interaction_ < 5.0 × 10^−8^. As the interaction studies of *SNP–SNP* or *SNP*–environment suffer greater power limitations compared to main effect SNP studies, there is a greater chance that variants of potential biological significance may be missed (type II error). We accordingly applied a liberal threshold of *P*_interaction_ < 1.0 × 10^−5^ to identify any SNPs of potential biological significance (referred below as “potentially relevant SNPs”). For the replication, we highlighted SNPs with marginal significance (*P*_interaction_ < 0.05). A *P*_interaction_ < 0.006 was required to meet the statistically significant threshold for testing of eight variants in the replication data using Bonferroni correction. Odds ratios (ORs) for the *APOL1* association with ESKD are presented for those with and without at least one alternate allele of the GWAS SNP of interest. Gene annotation was completed using ANNOVAR (23). Manhattan and QQ plots for *P*_interaction_ terms were generated using the R package qqman (24).

Because previously published studies were conducted among cases with non-diabetic nephropathy, we examined significant SNPs from discovery, by adjusting for diabetes in a sensitivity analysis. We further tested the associations in a sub-group of REGARDS participants who did not have diabetes at baseline. Finally, we report the estimates from both the discovery and replication cohorts for 14 SNPs that were statistically significant in earlier *APOL1*–SNP interaction studies for comparison (5, 6).

## Results

Among 8,074 participants in the REGARDS study, 3,357 had zero *APOL1* risk alleles, 3,697 had one risk allele, and 1,020 (13%) had two risk alleles. Similarly, the GenHAT study had 871 participants with two *APOL1* risk alleles, accounting for 13% of the cohort. The mean age of participants was 63.6 years (SD = 9.2) in REGARDS and 66.1 years (SD = 7.7) in GenHAT. Participants in the REGARDS study were less likely to be male compared to GenHAT (Table 1). While all the participants in GenHAT had a diagnosis of hypertension, 71% of REGARDS participants had a diagnosis of hypertension. The GenHAT inclusion criteria required participants to have hypertension and at least one other cardiovascular risk factor, and so the prevalence of diabetes was higher in GenHAT compared to REGARDS. Participants in the BioVU study were younger [mean (SD) = 47.0 (17.0)] compared to other studies. Characteristics of the BioVU study population can be found in Supplementary Table 1.

**Table 1:** Baseline characteristics of discovery and replication cohort.

	**REGARDS**	**GenHAT**
	**(discovery)**	**(replication)**
Mean (SD) or *N* (%)	8,074	6,791
Age	63.6 (9.2)	66.1 (7.7)
Male	3,177 (39.4)	3,028 (44.6)
Diabetes at baseline	2,330 (29.2)	4,063 (59.8)
Hypertension at baseline	5,738 (71.2)	6,791 (100)
Incident ESKD	388 (4.8)	128 (1.9)
*APOL1* risk alleles		
0	3,357 (41.6)	3,032 (44.6)
1	3,697 (45.7)	2,888 (42.5)
2	1,020 (12.6)	871 (12.8)

ESKD, end-stage kidney disease; REGARDS, REasons for Geographic and Racial Difference in Stroke; GenHAT, Genetics of Hypertension Associated Treatment.

**Figure 1:**
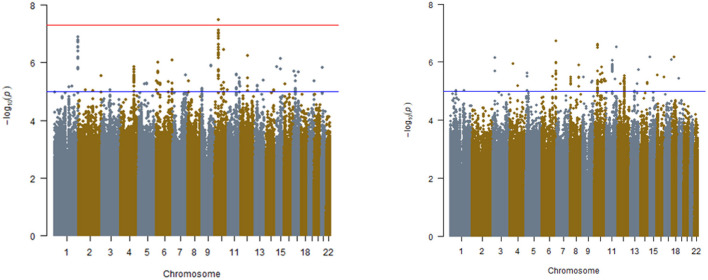
Manhattan plots for interaction analyses in REGARDS study. **(Left)** *APOL1* risk status determined by additive model, **(Right)** *APOL1* risk status determined by recessive model. Models include *APOL1*, SNP, APOL1*SNP, age, sex, and ancestry. The red line is indicative of genome-wide significance *p* ≤ 5.00 × 10^−08^. Blue line is indicative of suggestive significance, *p* ≤ 1.00 × 10^−05^.

Manhattan and QQ Plots discovery analysis in REGARDS are presented in Figure 1 (Left and Right panels) and Supplementary Figure 1. Only one variant (rs7067944) on chromosome 10 (~10 kB from the *PCAT5* gene) interacted with *APOL1* under additive inheritance with statistical significance after correcting for multiple testing (*P*_interaction_ = 3.4 × 10^−8^). Two SNPs in the same region almost met the statistical significance threshold (Table 2). None of these top SNPs were replicated in the GenHAT study. A total of 183 SNPs interacted with *APOL1* risk status under the additive genetic model with *P*_interaction_ < 1.0 × 10^−5^ for incident ESKD. Two loci of interest in REGARDS included *RNLS* (rs536243, *P*_interaction_ = 3.6 × 10^−7^) and *SYMD3* (rs75431828, *P*_interaction_ = 1.3 × 10^−7^) but these loci did not replicate in GenHAT (*P*_interaction_ > 0.05). See Supplementary File 1 Dataset S1 for the 183 top results in REGARDS along with the corresponding replication in GenHAT. When considering *APOL1* under a recessive genetic inheritance model, none of the variants met the genome-wide statistical significance threshold. However, 147 SNPs interacted with *APOL1* risk status with *P*_interaction_ < 1.0 × 10^−5^ for incident ESKD. Among these, the top hit for the recessive model was an intronic variant on the *SMOC2* gene located at chromosome 6 (rs62423404, *p*-interaction = 1.88 × 10^−7^); this was a region of interest identified in the *APOL1* additive inheritance model.

**Table 2:** Top APOL1–SNP interaction effects on ESKD in the REGARDS and GenHAT studies under *APOL1* additive model.

**Gene**	**Region**	**rsID**	**Chr**	**BP (hg38)**	**R**	**A**	**EAF**	**REGARDS**			**GenHAT**		
***APOL1*** **additive**								***APOL1*** **OR (95%CI)**		*P*_interaction_	***APOL1*** **OR (95%CI)**		*P*_interaction_
								≥**1 GWAS SNP minor alleles**	**0 GWAS SNP MINOR Alleles**		≥**1 GWAS SNP minor alleles**	**0 GWAS SNP MINOR Alleles**	
**Statistically significant SNPs**													
*PCAT5;ANKRD30A*	Intergenic	*rs7067944*	chr10	35810852	A	G	0.54	1.23 (0.90, 1.67)	3.19 (1.99, 5.11)	3.4E-08	1.48 (1.10, 1.99)	0.93 (0.60, 1.55)	0.45
*PCAT5;ANKRD30A*	Intergenic	*rs744372*	chr10	35809696	C	G	0.50	1.29 (0.96, 1.74)	3.11 (1.89, 5.15)	7.5E-08	1.44 (1.08, 1.93)	0.94 (0.54, 1.62)	0.68
*PCAT5;ANKRD30A*	Intergenic	*rs7086402*	chr10	35811113	T	C	0.54	1.28 (0.95, 1.75)	3.11 (1.91, 5.17)	8.9E-08	1.45 (1.08, 1.93)	0.85 (0.48, 1.50)	0.56
**Potentially relevant SNPs**													
*SMOC2*	Intronic	*rs62323403*	chr6	168647687	G	A	0.05	2.38 (1.78, 3.45)	1.03 (0.86, 1.22)	8.0E-07	1.66 (0.97, 2.83)	1.22 (0.91, 1.64)	0.27
*SMOC2;LOC105378146*	Intergenic	*rs2181251*	chr6	168669997	T	C	0.19	2.27 (1.53, 3.37)	1.34 (0.96, 1.85)	5.3E-06	1.83 (1.19, 2.81)	1.09 (0.80, 1.56)	0.07
*SMOC2;LOC105378146*	Intergenic	*rs62423451*	chr6	168672559	A	T	0.11	2.96 (1.78, 4.90)	1.34 (1.00, 1.80)	8.8E-06	2.10 (1.19, 3.71)	1.16 (0.87, 1.54)	0.20
*SMOC2;LOC105378146*	Intergenic	*rs11751195*	chr6	168672766	T	C	0.11	2.96 (1.78, 4.90)	1.34 (1.00, 1.80)	8.9E-06	2.10 (1.19, 3.71)	1.16 (0.87, 1.54)	0.20
*SMOC2;LOC105378146*	Intergenic	*rs4286744*	chr6	168672106	A	G	0.11	2.96 (1.78, 4.88)	1.34 (1.00, 1.80)	8.6E-06	2.10 (1.19, 3.71)	1.16 (0.87, 1.54)	0.28

Adjusted for age, sex, and principal components of ancestry; R, reference allele; A, alternate allele; EAF, effect allele frequency; chr, chromosome.

Among 183 SNPs identified in the discovery analysis for an additive inheritance, only five had a consistent direction of the interaction term in the GenHAT study (Table 2, Supplementary Table 2). Of those five SNPs (one intronic on SMOC2 gene, and four intergenic between *SMOC2* and *LOC105378146* on chromosome 6), rs62423403 was the most statistically significant (REGARDS *P*_interaction_ =8.0 × 10^−6^) and rs2181251 had the most consistent magnitude of association in both studies. Carriers of the alternate allele at rs2181251 variant had increased risk for ESKD associated with *APOL1* (OR [95%CI]: REGARDS: 2.27 [1.53, 3.37]; GenHAT: 1.83 [1.19, 2.81]), while non-carriers did not have elevated risk associated with *APOL1* (OR [95%CI] = 1.34 [0.96, 1.85]). The relationship was also similar for the other SNPs (Table 2). The associations for potentially-relevant variants of biological significance were similar for the *APOL1* recessive model in both REGARDS and GenHAT studies (Table 3).

**Table 3:** Top APOL1–SNP interaction effects on ESKD in the REGARDS and GenHAT studies under *APOL1* recessive model.

**Gene**	**Region**	**rsID**	**Chr**	**BP** ** (hg38)**	**R**	**A**	**EAF**	**REGARDS**			**GenHAT**		
***APOL1*** **recessive**								***APOL1*** **OR** **(95%CI)**		*P*_interaction_	***APOL1*** **OR** **(95%CI)**		*P*_interaction_
								≥**1 GWAS SNP minor alleles**	**0 GWAS SNP minor alleles**		≥**1 GWAS SNP minor alleles**	**0 GWAS SNP minor alleles**	
**Potentially relevant SNPs**													
*SMOC2*	Intronic	*rs62323403*	chr6	168647687	G	A	0.05	4.38 (2.70, 7.12)	1.00 (0.70, 1.42)	1.8E-07	2.10 (0.86, 5.11)	1.38 (0.81, 2.35)	0.37
*SMOC2;LOC105378146*	Intergenic	*rs2181251*	chr6	168669997	T	C	0.19	2.27 (1.53, 3.37)	0.93 (0.62, 1.39)	8.5E-06	1.93 (0.93, 4.01)	1.28 (0.71, 2.30)	0.25
*SMOC2;LOC105378146*	Intergenic	*rs62423451*	chr6	168672559	A	T	0.11	2.96 (1.78, 4.90)	1.05 (0.73, 1.49)	1.1E-06	2.77 (1.10, 6.96)	1.26 (0.74, 2.15)	0.30
*SMOC2;LOC105378146*	Intergenic	*rs11751195*	chr6	168672766	T	C	0.11	2.96 (1.78, 4.90)	1.05 (0.73, 1.49)	1.1E-06	2.77 (1.10, 6.96)	1.26 (0.74, 2.15)	0.30
*SMOC2;LOC105378146*	Intergenic	*rs4286744*	chr6	168672106	A	G	0.11	2.96 (1.78, 4.90)	1.01 (0.7, 1.43)	1.1E-06	2.84 (1.13, 7.14)	1.25 (0.74, 2.13)	0.35

Adjusted for age, sex, and principal components of ancestry; R, reference allele; A, alternate allele; EAF, effect allele frequency; Chr, chromosome.

In the sensitivity analysis adjusting for diabetes status, the *APOL1–*SNP interactions for the four SNPs near *SMOC2* were consistent with the primary analysis in REGARDS as well as in the GenHAT replication cohort (Supplementary Tables 3, 4). Results were also consistent when the analysis was restricted to participants without diabetes in each study (Supplementary Tables 3, 4). Although the direction and strength of the associations between *APOL1* risk variants and ESKD were consistent among those with at least one minor allele of SNP of interest, the *p*-interaction value for SNPs did not replicate in the BioVU study (Supplementary Tables 5, 6). None of the SNPs from prior studies were replicated in our cohort (Supplementary Tables 7, 8).

## Discussion

In this study of *APOL1* by gene interaction in community-dwelling AA with incident ESKD, we found a potential modifier locus that increased the risk of ESKD. *APOL1* risk status was significantly associated with incident ESKD among carriers but not among non-carriers of a minor allele at five SNPs near *SMOC2*. The findings were consistent with those in GenHAT (another large population of AA from a clinical trial of anti-hypertensive agents), but in GenHAT the interaction term did not meet the criteria for statistical significance. These variants are of interest due to the role of *SMOC2* in kidney injury. Further, findings from previous studies of *APOL1*-by-SNP interaction were not significant among participants without diabetes in the present study.

The primary SNPs identified in REGARDS (*rs2181251, rs62423451, rs11751195, rs4286744*) were in an intergenic region 2–5 kb downstream from the *SMOC2* gene. Additionally, another variant of interest (*rs62423403*) was intronic in the protein-coding transcript of the *SMOC2* gene. The relationship of *APOL1* with ESKD was heightened in the minor allele carriers of these SNPs. The relationship between *APOL1* and ESKD was OR [95% CI] = 4.38 (2.70, 7.12) for rs62323403 minor allele carriers vs. 1.00 (0.70, 1.42) for the common variant under the recessive model in REGARDS. Though potentially artifactual due to the restricted sample, these carrier differences were strongest among REGARDS participants without diabetes at baseline. For example, in our sensitivity analysis the *APOL1* OR [95% CI] for rs62423451 carriers was 8.2 [4.1, 16.5] and for non-carriers was 1.35[0.78, 2.35].

*SMOC2* (SPARC-related modular calcium binding 2) is a protein-coding gene influencing growth factor signaling, migration, proliferation, and angiogenesis (25, 26). SMOC2 protein is upregulated in renal tubular epithelial cells of kidney biopsies showing pathological fibrosis and SMOC2 plays a role in the progression of fibrosis (27). *SMOC2* is overexpressed in cell culture in response to angiotensin II-related progression of podocyte injury (28). APOL1 protein may not be expressed in tubular cells and filtered APOL1 could potentially interact with SMOC2 protein in podocytes inducing injury and subsequent proteinuria. In our analyses, the interaction effect between *SMOC2* and *APOL1* was present in absence of a main effect of the SNPs near *SMOC2*; this could explain the lack of data on this gene in a single-locus analysis. Still detecting a physical interaction is beyond the scope of population genetic studies such as ours. Additional population studies of ESKD among AA with and without diabetes are warranted.

We identified two loci, *RNLS* (encoding renalase) and *SMYD3* (SET and MYND domain containing 3), that are associated with kidney disorders in the REGARDS study but did not replicate in the GenHAT study. The *RNLS* renalase gene has been associated with ESKD and chronic kidney disease in prior studies (7, 29, 30). The gene codes for a protein secreted by the kidney which decreases systemic blood pressure in response to an increase in blood pressure or release of catecholamines, and regulates cardiovascular function (31). As renalase expression is associated with chronic kidney disease, the interesting possibility that *APOL1* interacts with this pathway requires further investigation. Additionally, *SMYD3* may contribute to autosomal dominant polycystic kidney disease and renal carcinoma via its lysine methyltransferase activity (32, 33). The variants that met the genome-wide statistical significance threshold for the *APOL*1^*^SNP interaction were in the intergenic region of *PCAT5* and *ANKRD30A* genes which have not previously been linked to kidney function and further characterization are warranted.

Differences between our study and prior gene modifier studies of *APOL1*-renal risk are that other studies focused on SNPs with main effects on ESKD as well as data from participants with non-diabetic ESKD. Bostrom et al. and Divers et al. examined a similar set of ESKD-related SNPs highlighting potential *APOL1* modifier variants in *NPHS2, SDCCAG8*, and *BMP4* (all known nephropathy loci) (5–7, 34, 35). In another study that used a case-only design, *APOL1* risk genotypes were associated with a variant, rs79741405 (base pair: 160673237), on chromosome 6 but this locus was far from the SNPs we identified on chromosome 6 in our study (7). Unfortunately, none of these variants were replicated in our study. One possible explanation could be the broader ESKD definition used in our study. However, our results did not change after adjusting our analyses for diabetes and/or restricting to those without diabetes at baseline.

A strength of our study was the use of more contemporary genotype data enriched for additional African variant coverage in three large cohorts of AA which allowed us to better define population substructure and test for additional variants. The identification of incident events from prospective cohort studies may have reduced selection bias as non-ESKD controls are representative of the population that produced the cases. Being a population-based study, there are always chances of measurement bias. Specifically, we could not discern the specific type of nephropathy or underlying cause associated with ESKD diagnosis in our cohorts. Better characterizing of phenotypes in our study as well as harmonizing of the phenotypes with prior studies could have improved our consistency with other published studies. BioVU study is an EHR-based study, and the population was younger compared to REGARDS and GenHAT. We did not test whether the interaction effects are age-dependent but there is a chance that biological mechanisms driven by these variants could be age-related. We cannot rule out the possibility that a higher rate of incident ESKD could also be a proxy for improved survival of CKD. APOL1 high-risk genotypes are associated with better survival after accounting for kidney-related comorbidities and genetic ancestry (36). However, high-risk APOL1 status has been associated with the progression of kidney function to ESKD among those with chronic kidney disease (37). Due to lack of multiple time-point measurements in our cohorts, we cannot discern these differences between improved survival and progression of CKD. While we found biologically relevant associations, we cannot rule out the possibility of type II error due to the underestimation of interaction effects in the standard regression-based interaction tests or lack of replication (38). However, the direction of the associations was consistent in the replication study which asserts the significance of these findings to a certain extent. Further translational work and well-powered studies can help determine if these associations are valid. *APOL1* is located at the Chromosome 22 locus which is enriched for intrachromosomal duplications and duplicated *APOL1* genotype segments with apparent risk genotypes have been observed in a few samples of 1,000 Genome population (39). Identifying such duplicated segments in our study population was beyond the scope of our study.

In conclusion, using a large GWAS effort in an AA population, we found that SNPs near the *SMOC2* gene had a significant interaction with *APOL1* in determining the risk of ESKD. In particular, *APOL1* was associated with a higher risk of ESKD in the presence of alternate alleles at those SNPs. The findings could help improve our understanding of the potential modifiers of *APOL1* risk status that contribute to the observed incomplete penetrance of that locus.

## Data availability statement

Publicly available datasets were analyzed in this study. This data can be found here: The raw REGARDS genotype and phenotype data used in this study can be found in dbGaP accession number phs002719.v1.p1. The raw GenHAT genotypic and phenotypic data used in this study are deposited in the National Center for Biotechnology Information (NCBI) Database for Genotypes and Phenotypes (dbGaP), accession number phs002716.v1.p1. The genotypes and phenotypes from BioVU are available to researchers who meet the criteria for access to confidential data, upon request and clearance from Vanderbilt University Medical Center Institutional Review Board and BioVU. Interested and eligible researchers may contact the BioVU data access team at biovu@vanderbilt.edu for more detailed information regarding access to phenotype data from BioVU.

## Ethics statement

The studies involving human participants were reviewed and approved by University of Alabama at Birmingham Institutional Review Board. The patients/participants provided their written informed consent to participate in this study.

## Author contributions

NC, MI, OG, HT, BH, NL, and RR: contributed to the study design. MI, OG, CW, and JK: oversaw the genotyping performed for the parent study. NC, NA, JH, HT, and MI: data analysis and interpretation. SJ, MI, and LL: parent study participant recruitment and data collection. MI, JH, and DA: replication study, participant, data collection, and access. NC, HT, and MI: first draft preparation. NC, NA, MI, OG, HT, BH, NL, RR, CW, JK, SJ, GN, LL, DA, and JH: critical inputs on the manuscript. All authors contributed important intellectual content during manuscript drafting or revision and agrees to be personally accountable for the individual's contributions and to ensure that questions pertaining to the accuracy or integrity of any portion of the work, even one in which the author was not directly involved, are appropriately investigated and resolved.

## Funding

This study was supported by the National Institutes of Health (NIH) National Heart, Lung, and Blood Institute (NHLBI) grants R01HL123782 (MI) and R01HL136666 (MI and LL). The research project is supported by cooperative agreement U01 NS041588 co-funded by the National Institute of Neurological Disorders and Stroke (NINDS) and the National Institute on Aging (NIA), National Institutes of Health, Department of Health and Human Service. JK was supported by the Intramural Research Program of National Institute of Diabetes and Digestive and Kidney Diseases (NIDDK). CW was supported in part by the National Institutes of Health and the National Cancer Institute Intramural Research Program and under contract HHSN26120080001E. NC was supported by American Heart Association Predoctoral Fellowship (AHA Award #18PRE34000021). GN was supported by R01DK127139. JH was supported by K12 HD04348. The dataset(s) used for the analyses described were obtained from Vanderbilt University Medical Center's BioVU which is supported by numerous sources: institutional funding, private agencies, and federal grants. These include the NIH-funded Shared Instrumentation Grant S10RR025141; and CTSA grants UL1TR002243, UL1TR000445, and UL1RR024975. Genomic data are also supported by investigator-led projects that include U01HG004798, R01NS032830, RC2GM092618, P50GM115305, U01HG006378, U19HL065962, R01HD074711, and additional funding sources listed at https://victr.vumc.org/biovu-funding/. Representatives from American Heart Association did not have any role in the design and conduct of the study, the collection, management, analysis, interpretation of the data, and the preparation or approval of the manuscript.

## Conflict of interest

The authors declare that the research was conducted in the absence of any commercial or financial relationships that could be construed as a potential conflict of interest.

## Publisher's note

All claims expressed in this article are solely those of the authors and do not necessarily represent those of their affiliated organizations, or those of the publisher, the editors and the reviewers. Any product that may be evaluated in this article, or claim that may be made by its manufacturer, is not guaranteed or endorsed by the publisher.

## Author disclaimer

The content is solely the responsibility of the authors and does not necessarily represent the official views of the NINDS or the NIA.

## Supplementary material

Supplementary Figure 1 QQ plots for interaction analyses in the REGARDS study (corresponding to Figure 1 Manhattan plots). **(Left)** *APOL1* risk status determined by additive model; **(Right)** *APOL1* risk status determined by the recessive model. Models include *APOL1*, SNP, *APOL*1^*^SNP, age, sex, and ancestry.
