# Supplementary material for: SMOC2 gene interacts with APOL1 in the development of end-stage kidney disease: A genome-wide association study
Source: Front Med (Lausanne). 2022 Sep 28;9:971297. doi: 10.3389/fmed.2022.971297 (PMC9554233; doi:10.3389/fmed.2022.971297)

***SMOC2* Gene interacts with *APOL1* in the development of end-stage kidney disease: a genome-wide association study**

Ninad S Chaudhary, Nicole D Armstrong, Bertha A Hidalgo, Orlando M Gutiérrez, Jacklyn N Hellwege, Nita A Limdi, Richard J Reynolds, Suzanne E Judd, Girish N Nadkarni, Leslie Lange, Cheryl A Winkler, Jeffrey B Kopp, Donna K Arnett, Hemant K Tiwari, Marguerite R Irvin

Supplemental Table 1: Baseline characteristics of the BioVU study

	**BioVU**
**Mean (SD) or N(%)**	
**Total**	9,566
**Age**	47 (17)
**Male**	3,300 (34.91%)
**Diabetes at baseline**	1,713 (19.64%)
**Hypertension at baseline**	6,701 (70.05%)
**Incident ESKD**	
**Yes**	632
**No**	8,934
***APOL1* risk alleles**	
**0**	3,875 (40.51%)
**1**	4,289 (44.84%)
**2**	1,402 (14.66%)

ESKD = Eng Stage Kidney Disease; REGARDS = REasons for Geographic and Racial Difference in Stroke; GenHAT = Genetics of Hypertension Associated Treatment

Supplemental Table 2: Pairwise LD (R^2) for potentially relevant SNPs

	rs62323403	*rs2181251*	*rs62423451*	*rs11751195*	*rs4286744*
rs62323403	--				
rs2181251	0.30	--			
rs62423451	0.99	0.24	--		
rs11751195	0.32	0.24	1	--	
rs4286744	0.32	0.24	0.99	0.99	--

Supplementary Table 3: Top *APOL1*-SNP interaction effects on ESKD in the REGARDS and GenHAT studies accounting for diabetes under additive inheritance

Gene	Region	rsID	Chr	BP(hg38)	R	A	EAF	REGARDS			GenHAT		
***APOL1* Additive**								***APOL1* OR(95%CI)**		**P interaction**	***APOL1* OR(95%CI)**		**P interaction**
								**≥ 1 Minor Allele**	**0 minor Allele**		**≥ 1 Minor Allele**	**0 minor Allele**	
***Adjusting for diabetes*[a]**													
**Statistically Significant**													
PCAT5;ANKRD30A	intergenic	*rs7067944*	chr10	35810852	A	G	0.54	0.99(0.82,1.19)	2.23(1.68,2.97)	2.5E-07	1.46(1.09,1.97)	0.92(0.55,1.54)	0.42
PCAT5;ANKRD30A	intergenic	*rs744372*	chr10	35809696	C	G	0.50	1.00(0.83,1.20)	2.36(1.74,3.18)	7.6E-07	1.43(1.07,1.91)	0.91(0.52,1.69)	0.63
PCAT5;ANKRD30A	intergenic	*rs7086402*	chr10	35811113	T	C	0.54	1.00(0.82,1.19)	2.37(1.75,3.19)	9.6E-07	1.43(1.08,1.91)	0.83(0.46,1.47)	0.50
**Potentially Relevant SNPs**													
SMOC2	intronic	*rs62323403*	chr6	168647687	G	A	0.05	2.52(1.79,3.53)	1.04(0.87,1.24)	2.1E-06	1.62(0.95,2.77)	1.21(0.91,1.62)	0.28
SMOC2;LOC105378146	intergenic	*rs2181251*	chr6	168669997	T	C	0.19	1.93(1.50,2.47)	0.92(0.61,1.37)	3.9E-05	1.81(1.18,2.78)	1.08(0.79,1.49)	0.08
SMOC2;LOC105378146	intergenic	*rs62423451*	chr6	168672559	A	T	0.11	2.34(1.68,3.24)	1.06(0.89,1.26)	1.6E-06	2.08(1.16,3.73)	1.16(0.87,1.53)	0.20
SMOC2;LOC105378146	intergenic	*rs11751195*	chr6	168672766	T	C	0.11	2.33(1.68,3.24)	1.06(0.89,1.26)	1.6E-06	2.11(1.18,3.77)	1.16(0.87,1.54)	0.20
SMOC2;LOC105378146	intergenic	*rs4286744*	chr6	168672106	A	G	0.11	2.34(1.68,3.24)	1.06(0.89,1.26)	1.6E-06	2.08(1.16,3.73)	1.16(0.87,1.53)	0.29
***Among those without diabetes*[b]**													
**Statistically Significant**													
PCAT5;ANKRD30A	intergenic	*rs7067944*	chr10	35810852	A	G	0.54	1.23(0.90,1.67)	3.19(1.99,5.11)	0.0004	1.45(1.00.2.09)	1.12(0.54,2.31)	0.18
PCAT5;ANKRD30A	intergenic	*rs744372*	chr10	35809696	C	G	0.50	1.29(0.96,1.74)	3.11(1.90,5.10)	0.0008	0.80(0.40,1.63)	1.22(0.59,2.53)	0.22
PCAT5;ANKRD30A	intergenic	*rs7086402*	chr10	35811113	T	C	0.54	1.28(0.95,1.73)	3.11(1.91,5.07)	0.0012	0.80(0.40,1.68)	1.22(0.59,2.53)	0.22
**Potentially Relevant SNPSs**													
SMOC2	intronic	*rs62323403*	chr6	168647687	G	A	0.05	4.21(2.35,7.53)	1.31(0.99,1.75)	7.0E-04	1.69(0.89,3.22)	1.15(0.80,1.68)	0.20
SMOC2;LOC105378146	intergenic	*rs2181251*	chr6	168669997	T	C	0.19	2.27(1.53,3.37)	1.34(0.96,1.85)	0.03	1.74(1.00,3.03)	1.08(0.73,1.61)	0.32
SMOC2;LOC105378146	intergenic	*rs62423451*	chr6	168672559	A	T	0.11	2.96(1.78,4.90)	1.34(0.99,1.80)	1.0E-04	1.79(0.94,3.44)	1.11(0.77,1.61)	0.30
SMOC2;LOC105378146	intergenic	*rs11751195*	chr6	168672766	T	C	0.11	2.96(1.78,4.90)	1.34(0.99,1.80)	1.0E-04	1.79(0.94,3.44)	1.11(0.77,1.61)	0.30
SMOC2;LOC105378146	intergenic	*rs4286744*	chr6	168672106	A	G	0.11	2.96(1.78,4.90)	1.34(0.99,1.80)	1.0E-04	1.83(0.96,3.50)	1.11(0.77,1.61)	0.42

[a]adjusted for age, sex, principal components of ancestry and diabetes status; [b] adjusted for age, sex and principal components of ancestry, ;R= Reference allele, A = Alternate allele, EAF = effect allele frequency, Chr = Chromosome

Supplementary Table 4: Top *APOL1*-SNP interaction effects on ESKD in the REGARDS and GenHAT studies (*APOL1* recessive)

Gene	Region	rsID	Chr	BP(hg38)	R	A	EAF	REGARDS			GenHAT		
***APOL1* Recessive**								***APOL1* OR(95%CI)**		**P interaction**	***APOL1* OR(95%CI)**		**P interaction**
**Potentially Relevant SNPs**								**≥ 1 Minor Allele**	**0 minor Allele**		**≥ 1 Minor Allele**	**0 minor Allele**	
Adjusting for diabetes[a]													
SMOC2	intronic	*rs62323403*	chr6	168647687	G	A	0.05	4.54(2.33,7.55)	1.04(0.73,1.48)	4.1E-07	1.99(0.81,4.89)	1.35(0.79,2.31)	0.38
SMOC2;LOC105378146	intergenic	*rs2181251*	chr6	168669997	T	C	0.19	2.91(1.98,4.28)	0.92(0.61,1.37)	5.4E-06	1.88(0.90,3.89)	1.26(0.70,2.28)	0.27
SMOC2;LOC105378146	intergenic	*rs62423451*	chr6	168672559	A	T	0.11	4.25(2.65,6.82)	1.01(0.71,1.43)	1.6E-06	2.74(1.07,7.02)	1.24(0.73,2.11)	0.30
SMOC2;LOC105378146	intergenic	*rs11751195*	chr6	168672766	T	C	0.11	4.24(2.64,6.81)	1.01(0.72,1.43)	1.6E-06	2.74(1.07,7.01)	1.24(0.73,2.11)	0.30
SMOC2;LOC105378146	intergenic	*rs4286744*	chr6	168672106	A	G	0.11	4.41(2.71,7.17)	1.05(0.73,1.49)	1.6E-06	2.83(1.11,7.26)	1.23(0.72,2.10)	0.34
Among those without diabetes[b]													
SMOC2	intronic	*rs62323403*	chr6	168647687	G	A	0.05	9.67(4.53,21.94)	1.45(0.86,2.46)	6.4E-05	1.94(0.65,5.76)	1.14(0.55,2.36)	0.29
SMOC2;LOC105378146	intergenic	*rs2181251*	chr6	168669997	T	C	0.19	4.19(2.21,7.61)	1.62(0.91,2.89)	0.01	1.85(0.71,4.78)	1.06(0.49,2.30)	0.41
SMOC2;LOC105378146	intergenic	*rs62423451*	chr6	168672559	A	T	0.11	8.19(4.06,16.54)	1.35(0.78,2.35)	9.9E-05	2.07(0.70,6.19)	1.06(0.51,2.19)	0.39
SMOC2;LOC105378146	intergenic	*rs11751195*	chr6	168672766	T	C	0.11	8.19(4.06,16.55)	1.36(0.78,2.35)	0.0001	2.07(0.69,6.18)	1.06(0.51,2.19)	0.39
SMOC2;LOC105378146	intergenic	*rs4286744*	chr6	168672106	A	G	0.11	8.20(4.06,16.56)	1.35(0.78,2.35)	9.7E-05	2.18(0.73,6.50)	1.05(0.51,2.17)	0.44

[a]adjusted for age, sex, principal components of ancestry and diabetes status; [b] adjusted for age, sex and principal components of ancestry, ;R= Reference allele, A = Alternate allele, EAF = effect allele frequency, Chr = Chromosome

Supplemental Table 5: ***APOL1*-SNP interaction effects on ESKD in the BioVU study under an *APOL1* additive model**

Gene	Region	rsID	Chr	BP(hg38)	R	A	EAF	BioVU		
***APOL1* Additive**								***APOL1* OR(95%CI)**		**P interaction**
								**≥ 1 GWAS SNP Minor Alleles**	**0 GWAS SNP minor Alleles**	
**Statistically Significant**										
PCAT5;ANKRD30A	intergenic	*rs7067944*	chr10	35810852	A	G	0.54	2.00 (1.74-2.29)	2.14 (1.71-2.68)	0.82
PCAT5;ANKRD30A	intergenic	*rs744372*	chr10	35809696	C	G	0.50	1.96 (1.71-2.24)	2.29 (1.80-2.90)	0.84
PCAT5;ANKRD30A	intergenic	*rs7086402*	chr10	35811113	T	C	0.54	1.97 (1.72-2.25)	2.26 (1.78-2.87)	0.79
**Potentially Relevant SNPs**										
SMOC2	intronic	*rs62323403*	chr6	168647687	G	A	0.05			NA
SMOC2; LOC105378146	intergenic	*rs2181251*	chr6	168669997	T	C	0.19	1.87 (1.54-2.25)	2.13 (1.83-2.47)	0.53
SMOC2; LOC105378146	intergenic	*rs62423451*	chr6	168672559	A	T	0.11	1.97 (1.53-2.53)	2.06 (1.80-2.35)	0.54
SMOC2; LOC105378146	intergenic	*rs11751195*	chr6	168672766	T	C	0.11	1.99 (1.55-2.55)	2.05 (1.79-2.34)	0.55
SMOC2; LOC105378146	intergenic	*rs4286744*	chr6	168672106	A	G	0.11	1.97 (1.53-2.53)	2.06 (1.80-2.35)	0.54

adjusted for age, sex, and principal components of ancestry;R= Reference allele, A = Alternate allele, EAF = effect allele frequency, chr = Chromosome

Supplemental Table 6: *APOL1*-SNP interaction effects on ESKD in the BioVU study under an *APOL1* recessive model

Gene	Region	rsID	Chr	BP(hg38)	R	A	EAF	BioVU		
***APOL1* Recessive**								***APOL1* OR(95%CI)**		**P interaction**
								**≥ 1 GWAS SNP Minor Alleles**	**0 GWAS SNP minor Alleles**	
**Potentially Relevant SNPs**										
SMOC2	intronic	*rs62323403*	chr6	168647687	G	A	0.05			
SMOC2;LOC105378146	intergenic	*rs2181251*	chr6	168669997	T	C	0.19	3.30 (2.47-4.42)	4.00 (3.18-5.01)	0.58
SMOC2;LOC105378146	intergenic	*rs62423451*	chr6	168672559	A	T	0.11	3.27 (2.22-4.82)	3.87 (3.16-4.74)	0.43
SMOC2;LOC105378146	intergenic	*rs11751195*	chr6	168672766	T	C	0.11	3.33 (2.26-4.89)	3.86 (3.15-4.72)	0.46
SMOC2;LOC105378146	intergenic	*rs4286744*	chr6	168672106	A	G	0.11	3.28 (2.22-4.82)	3.87 (3.16-4.73)	0.44

adjusted for age, sex, and principal components of ancestry;R= Reference allele, A = Alternate allele, EAF = effect allele frequency, chr = Chromosome

Supplemental Table 7: Estimates for incident ESKD of interaction between significant SNPs from prior studies and *APOL1* additive in REGARDS and GenHATparticipants without diabetes at baseline

Gene	Region	rsID	REGARDS			GenHAT		
			BETA	SE	p	BETA	SE	p
BUB3;GPR26	intergenic	rs7897598	-0.23	0.18	0.21	-0.09	0.12	0.48
HTR2A;LINC00562	intergenic	rs7986369	-0.03	0.18	0.85	-0.03	0.12	0.83
TUBGCP3;ATP11AUN	intergenic	rs2317446	0.39	0.21	0.07	-0.30	0.16	0.06
MIR4307HG;LINC00645	intergenic	rs12587505	-0.17	0.18	0.33	0.06	0.12	0.60
BMP4;CDKN3	intergenic	rs8014363	-0.03	0.19	0.86	-0.11	0.13	0.38
PLA2G4E	intronic	rs1668565	0.15	0.18	0.40	0.04	0.12	0.73
FOXB1;ANXA2	intergenic	rs6494167	0.25	0.18	0.17	0.10	0.12	0.39
NPHS1	exonic	rs437168	0.48	0.18	0.007	0.19	0.12	0.12
NPHS1	exonic	rs392702	0.51	0.20	0.01	0.34	0.15	0.02
NPHS2	intronic	rs16854341	0.16	0.24	0.51	-0.12	0.16	0.44
SDCCAG8	intronic	rs2802723	0.10	0.20	0.60	-0.21	0.14	0.15
FAM49A;RAD51AP2	intergenic	rs12988998	-0.25	0.18	0.16	0.05	0.12	0.66
LINC00471;NMUR1	intergenic	rs6754952	0.08	0.17	0.64	0.12	0.12	0.31
COBL;POM121L12	intergenic	rs7810220	-0.01	0.21	0.95	0.03	0.14	0.80
FAM3C;PTPRZ1	intergenic	rs10253361	0.25	0.20	0.21	0.12	0.14	0.37
STC1;ADAM28	intergenic	rs1586171	0.04	0.26	0.87	-0.17	0.16	0.26
DUSP26;LINC01288	intergenic	rs11776207	0.03	0.17	0.86	-0.22	0.12	0.07
TRIB1;LINC00861	intergenic	rs4457349	0.21	0.17	0.22	-0.09	0.12	0.45

Models adjusted for age, sex, and principal components of ancestry

Supplemental Table 8: Estimates for incident ESKD of interaction between significant SNPs from prior studies and *APOL1* recessive in REGARDS and GenHATparticipants without diabetes at baseline

Gene	Region	rsID	REGARDS			GenHAT		
			BETA	SE	p	BETA	SE	p
BUB3;GPR26	intergenic	rs7897598	-0.15	0.12	0.43	-0.15	0.11	0.17
HTR2A;LINC00562	intergenic	rs7986369	-0.15	0.13	0.23	0.03	0.10	0.76
TUBGCP3;ATP11AUN	intergenic	rs2317446	0.30	0.15	0.03	-0.12	0.13	0.34
MIR4307HG;LINC00645	intergenic	rs12587505	-0.10	0.15	0.50	0.03	0.10	0.78
BMP4;CDKN3	intergenic	rs8014363	-0.10	0.14	0.47	-0.12	0.11	0.29
PLA2G4E	intronic	rs1668565	0.11	0.12	0.37	0.01	0.10	0.92
FOXB1;ANXA2	intergenic	rs6494167	0.18	0.14	0.15	0.05	0.10	0.66
NPHS1	exonic	rs437168	0.15	0.12	0.19	0.12	0.10	0.23
NPHS1	exonic	rs392702	0.15	0.14	0.29	0.25	0.12	0.04
NPHS2	intronic	rs16854341	0.02	0.39	0.90	0.12	0.14	0.39
SDCCAG8	intronic	rs2802723	0.05	0.34	0.75	-0.10	0.13	0.46
FAM49A;RAD51AP2	intergenic	rs12988998	-0.22	0.15	0.11	0.03	0.10	0.76
LINC00471;NMUR1	intergenic	rs6754952	0.00	0.11	0.76	0.13	0.10	0.20
COBL;POM121L12	intergenic	rs7810220	-0.10	0.15	0.61	0.13	0.12	0.26
FAM3C;PTPRZ1	intergenic	rs10253361	-0.10	0.15	0.69	0.10	0.12	0.39
STC1;ADAM28	intergenic	rs1586171	0.04	0.17	0.71	-0.14	0.13	0.30
DUSP26;LINC01288	intergenic	rs11776207	-0.10	0.15	0.46	-0.10	0.11	0.34
TRIB1;LINC00861	intergenic	rs4457349	0.04	0.13	0.67	-0.07	0.10	0.51

Models adjusted for age, sex, and principal components of ancestry

Supplementary Figure 1: QQ Plots for Interaction Analyses in the REGARDS study (corresponding to Figure 1 Manhattan plots)

Footnote: Left Panel: *APOL1* risk status determined by additive model; Right Panel: *APOL1* risk status determined by the recessive model. models include *APOL1*, SNP, *APOL1SNP, age, sex, and ancestry.**

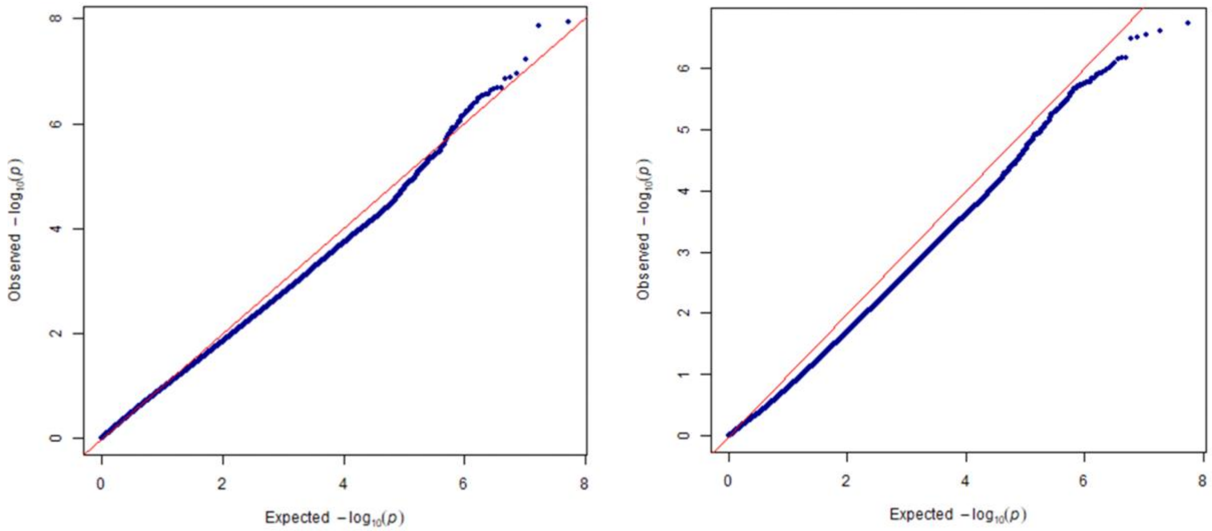

Supplement: Supplementary Figure 1 — QQ plots for interaction analyses in the REGARDS study (corresponding to Figure 1 Manhattan plots). (Left) APOL1 risk status determined by additive model; (Right) APOL1 risk status determined by the recessive model. Models include APOL1, SNP, APOL1*SNP, age, sex, and ancestry. [file Data_Sheet_2.pdf]
